# Role of *msbB* Gene in Physiology and Pathogenicity of *Vibrio parahaemolyticus*

**DOI:** 10.3390/microorganisms13020386

**Published:** 2025-02-10

**Authors:** Jinyuan Che, Binghong Liu, Qitong Fang, Shaojie Hu, Lei Wang, Baolong Bao

**Affiliations:** 1 Key Laboratory of Yangtze River Water Environment, Ministry of Education, College of Environmental Science and Engineering, Tongji University, Shanghai 200092, China; 22310015@tongji.edu.cn; 2 Key Laboratory of Exploration and Utilization of Aquatic Genetic Resources, Ministry of Education, National Demonstration Center for Experimental Fisheries Science Education, Shanghai Ocean University, Shanghai 201306, China; m230100053@st.shou.edu.cn (B.L.); 19853565191@163.com (Q.F.); 15233813696@163.com (S.H.)

**Keywords:** *V. parahaemolyticus*, *msbB* gene, virulence, shrimp infection, pathogenicity

## Abstract

The *msbB* gene, encoding a lipid A phosphatease, is crucial for lipopolysaccharide (LPS) synthesis in Gram-negative bacteria and plays a critical role in their virulence. This study investigated the role of *msbB* in *Vibrio parahaemolyticus*, a significant marine pathogen causing gastroenteritis in humans and infections in aquatic animals. We constructed an *msbB* deletion mutant (Δ*msbB*) and a complementary strain (CΔ*msbB*) using homologous recombination. The growth, outer membrane permeability, stress and antibiotic sensitivity, biofilm formation, swarming motility, and virulence of the wild-type (WT), Δ*msbB*, and CΔ*msbB* strains were assessed. Additionally, the pathogenicity of Δ*msbB* was evaluated using *L. vannamei* shrimp models. The results showed that the *msbB* gene was successfully deleted and complemented, and its deletion did not impair bacterial growth. However, the Δ*msbB* strain exhibited an increased outer membrane permeability, reduced resistance to stresses and antibiotics, defective biofilm formation, and a reduced swarming motility. In a *Tetrahymena* co-culture, the Δ*msbB* strain showed attenuated virulence. In shrimp infected with the Δ*msbB* strain, the cumulative mortality rate was 22%, significantly lower than the 62% observed in the WT strain. Moreover, the expression levels of immune-related genes in the shrimp hepatopancreas were significantly lower in the Δ*msbB* group, indicating a significant reduction in infection capability and pathogenicity. These findings indicate that the *msbB* gene is critical for the virulence of *V. parahaemolyticus* and suggest that *msbB* is a potential target for therapeutic interventions and vaccine development against *V. parahaemolyticus* infections.

## 1. Introduction

*Vibrio parahaemolyticus* is a Gram-negative, halophilic bacteria that is extensively found in aquatic environments [1,2,3,4]. It is a leading cause of seafood-borne gastroenteritis in humans, causing symptoms such as diarrhea, vomiting, and abdominal pain [5,6]. The bacteria is also a significant pathogen in aquaculture, particularly in shrimp farming, where it can lead to mass mortality and substantial economic losses.

Currently, *V. parahaemolyticus* has been extensively studied to elucidate the molecular mechanisms underlying its pathogenesis and interactions with host animals. It can produce various virulence factors to establish infection, evade host defenses, and cause disease [7,8,9,10]. Among these factors, toxins, in particular, are one of the most pivotal virulence factors in the pathogenicity of many bacterial species [8,11,12,13]. They have been the focus of extensive research due to their direct impact on host physiology. They can be broadly classified into two main categories, exotoxins and endotoxins, each with distinct characteristics and modes of action [14]. Exotoxins are heat-labile proteins that bacteria actively secrete into their surrounding environment. These proteins, such as thermostable direct hemolysin gene (*tdh*) and *tdh*-related hemolysin gene (*trh*), have been extensively studied in *V. parahaemolyticus*. Studies have been confirmed that the deletion of the *tdh* and *trh* genes results in a decreased hemolytic ability and attenuated pathogenicity [15]. In contrast, research on the virulence and endotoxins of *V. parahaemolyticus* is relatively limited. Endotoxins, which are heat-stable complexes composed of lipopolysaccharides and proteins, are essential components of the outer membrane in Gram-negative bacteria [16,17]. Lipopolysaccharide (LPS), a major constituent of this outer membrane, is well-known for its ability to trigger immune responses and induce inflammation in the host organism [11,18,19,20]. Attenuated vaccines developed through genetic modification or heat inactivation have shown some safety and protective efficacy against bacterial infections. Nevertheless, endotoxins exhibit a remarkable stability, allowing them to withstand extreme temperatures and pH levels [21,22]. Reducing endotoxin levels is a key determinant in ensuring the safety of attenuated live vaccines.

The active site of endotoxins is lipid A, which mediates almost all of the biological responses of endotoxins [23]. Lipid A is a crucial component of lipopolysaccharide (LPS), a major virulence factor in Gram-negative bacteria. Mutations in lipid A biosynthesis pathways often result in significant alterations to bacterial physiology and pathogenicity. Most mutations in lipid A biosynthesis result in a conditionally lethal phenotype [24]. In *V. parahaemolyticus*, the structure of lipid A is synthesized through the four following secondary acyltransferases: VP_RS00880, VP_RS08405, VP_RS12170, and VP_RS01045 (*msbB*) [25,26]. The simultaneous knockout of these four genes severely inhibits bacterial growth, highlighting the essential role of lipid A in bacterial viability. However, the deletion of any one of them only alters the structure of LPS without affecting bacterial growth [25], suggesting that each enzyme may have a specific role in fine-tuning the structure and function of LPS. Among these genes, the *msbB* gene, encoding a lipid A phosphatase, is particularly noteworthy. It is crucial for LPS synthesis in many Gram-negative bacteria. In *E. coli* and *N. meningitidis*, *msbB* deletion impairs lipid A synthesis, thereby affecting outer membrane integrity [27,28]. This highlights the central role of *msbB* in maintaining the structural integrity and functionality of LPS. Additionally, strains of *S. typhimurium* with the *msbB* gene deleted have been engineered to serve as potential anti-cancer therapies, demonstrating significant tumor-suppressing capabilities in mouse models [29]. This innovative application underscores the potential of manipulating *msbB* to alter bacterial properties for therapeutic purposes. Furthermore, *msbB* deletion affects host–immune interactions in a species-specific manner. LPS from *msbB*-deleted bacteria shows a reduced activation of Toll-like receptor 4 (TLR4), leading to a weakened inflammatory response and higher survival rates in infected mice [30]. Conversely, in *E. coli*, *msbB* deletion elicits a stronger adaptive immune response, aiding infection clearance [27]. Given its role in pathogenicity and immune modulation, *msbB* is a potential therapeutic target. Additionally, *msbB*-deleted bacteria or their LPS show promise as vaccine candidates [31]. Moreover, the *msbB* mutant of Salmonella exhibits reduced inflammatory risks, making it a safer option for vaccine development.

Despite these findings in other bacteria, the role of the *msbB* gene in *V. parahaemolyticus*, a significant marine pathogen causing gastroenteritis in humans and infections in aquatic animals such as shrimp, remains largely unexplored. In this study, we constructed an *msbB* deletion mutant strain to investigate its impact on the virulence of *V. parahaemolyticus* and the changes in pathogenicity following infection in shrimp.

## 2. Materials and Methods

### 2.1. Strains, Media, and Experimental Animals

The bacterial strains and plasmids used in this research are detailed in Table 1. The *V. parahaemolyticus* strain (ATCC^®^ 17802™, Guangdong Culture Collection Centre of Microbiology, Guangzhou, China) was specifically selected for the generation of deletion mutants and subsequent functional studies. Both *V. parahaemolyticus* and its derived mutants were cultivated in Luria–Bertani (LB) broth at a temperature of 30 °C, with continuous shaking at 150 rpm. The *Tetrahymena thermophila* strain was kindly provided by Prof. Shan Gao from the Ocean University of China. *T. thermophila* was maintained axenically in SPP medium, which comprised 2% proteose peptone, 0.1% yeast extract, 0.2% glucose, and 0.003% sequestrene, also at 30 °C. Healthy specimens of the white shrimp *L*. *vannamei* were sourced from Rufu Farm, located in Nantong, Jiangsu Province, China.

### 2.2. Construction of the msbB Deletion Mutant and Its Complementary Strain

The *msbB* deletion mutant (∆*msbB*) and its complementary strain were constructed via homologous recombination [32]. The primers used are listed in Table 2. The upstream (635 bp) and downstream (759 bp) flanking regions of *msbB* were amplified using the primers *msbB*-P1/P2 and *msbB*-P3/P4, respectively. These fragments were then ligated into the linearized suicide plasmid pSR47S, which had been digested with *BamH* I and *Sal* I, using the ClonExpress II One Step Cloning Kit (C112-01, Vazyme, Nanjing, China). The resulting pSR47S-∆*msbB* plasmid was transformed into CC118 λpir, then conjugated into the wild-type (WT) strain. Mutants were selected on LB agar with kanamycin (Kan) and ampicillin (Amp), and screened on LB agar with 10% sucrose. Mutation was confirmed by PCR and sequencing using the primers ∆*msbB* T1/T2. The complementary strain was constructed as previously reported [33]. A 2313 bp *msbB* fragment was amplified from the WT using the primers *msbB*-H1/H2, ligated into pSR47S, and transformed into ∆*msbB* via conjugation [33]. Successful complementation was verified by PCR using the primers C∆*msbB* T1/T2. Growth characteristics were evaluated by incubating cultures at 30 °C with shaking at 150 rpm until OD_600_ reached 1.0. The cultures were then diluted 1:100 and grown in a temperature-controlled incubator, with samples collected hourly.

### 2.3. Biofilm Formation

Biofilm formation was assessed using a modified method. Briefly, 20 µL of overnight cultures of the WT and Δ*msbB* strains were diluted 1:100 in fresh LB medium in a 96-well plate and incubated for 24 h at 30 °C. Biofilms were washed, stained with 0.1% crystal violet for 30 min, rinsed, and air dried. Then, 100 µL of 95% ethanol was added to solubilize the stain, and absorbance was measured at 595 nm. Each experiment was performed in triplicate.

### 2.4. Morphological Observation and Swarming Motility Analysis

For transmission electron microscopy (TEM) analysis, bacterial cultures were grown on 1% agar plates for 12 h at 30 °C. Subsequently, samples were negatively stained with 1% uranyl acetate and examined using a Tecnai G2F30 TEM (TEM, Tecnai G2F30, FEI Co., Hillsboro, OR, USA). For swarming motility assays, 2 µL of bacterial suspension (OD_600_ = 0.2) was spotted onto LB agar plates with 0.3% agar, following the protocol by Che et al. [32]. The plates were incubated at 30 °C for 12 h, and swarming motility was evaluated by measuring the diameter of the swarming zone. Each strain was tested in triplicate.

### 2.5. Outer Membrane Permeabilization Assay and Sensitivity Analysis for Stress and Antibiotics

For stress and antibiotic sensitivity analyses, the following stress factors were used: 2.5 mM M NaCl, 4 mM KH_2_PO_4_, and 0.04 mM H_2_O_2_. MIC assay was tested using antibiotics including β-lactams (cefotaxime and ampicillin), aminoglycosides (spectinomycin, gentamicin, streptomycin, tobramycin, and kanamycin), quinolone antibiotics (nalidixic aicd), macrolide antibiotics (clarithromycin), furanomycin, rifampicin, tetracycline, and chloramphenicol. The bacterial suspensions were inoculated into the stress factors and diluted antibiotic solutions (1:1 ratio) and incubated at 180 rpm and 30 °C for 24 h. Survival rate assays were performed on antibiotics that exhibited alterations in the stress factor and minimum inhibitory concentration (MIC), specifically ampicillin, nalidixic acid, clarithromycin, spectinomycin, and tobramycin. Overnight cultures were subcultured at a dilution of 1:100 in LB and incubated until the optical density at 600 nm (OD_600_ nm) reached 0.5. The cells were subsequently washed twice and resuspended in fresh LB. The cultures were then resuspended in either LB alone or LB supplemented with 4 mM KH_2_PO_4_, 2.5 M NaCl, 0.4 mM H_2_O_2_, 8 µg/mL ampicillin, 0.05 µg/mL nalidixic acid, 25 µg/mL clarithromycin, 6.25 µg/mL spectinomycin, and 1.25 µg/mL tobramycin. The cultures were incubated for 12 h at 30 °C in a rotary shaker. Following incubation, the OD_600_ of the cultures was measured using a BioTek Synergy HTX microplate reader (Agilent, Cheadle, UK).

### 2.6. Assessment of Strains’ Virulence Using Tetrahymena Model

To evaluate the virulence of the ∆*msbB* mutant, the *Tetrahymena* infection model was employed. This model assesses the relative survival rates of bacteria and *Tetrahymena* after co-culture, as previously reported [34]. *Tetrahymena* was adjusted to 1 × 10^5^ cells/mL using saline. The WT and ∆*msbB* mutants were adjusted to 3 × 10^9^ CFU/mL. Then, the *Tetrahymena* and bacterial strains were mixed at a 1:5000 ratio and co-cultured at 30 °C for 6 h. Bacterial growth was monitored hourly by measuring absorbance at 600 nm. Controls included bacterial strains mixed with saline and sterile saline as the blank. The relative survival rate of bacteria was calculated based on the number of bacteria remaining in co-culture compared to those grown alone.

For the virulence test of bacterial lysates on Tetrahymena, overnight bacterial cultures were adjusted to an OD_600_ of 1.0. Five milliliters of each culture was centrifuged at 10,000× *g* for 10 min to collect the cell pellets. The pellets were resuspended in 1 mL of saline and sonicated to prepare lysates. The lysates were mixed with Tetrahymena at a 1:1 volume ratio and incubated. After 6 h, CCK8 reagent was added to assess Tetrahymena viability (40203ES60, Yeasen, Shanghai, China).

### 2.7. Pathogenicity Assay

Shrimps were divided into three groups and were either infected with the WT strain or the ∆*msbB* mutant at a concentration of 4 × 10^7^ CFU/mL, or left uninfected as a control. The survival of the shrimp was tracked for 7 days after challenge, with mortality recorded. Relative percent survival (RPS, %) was calculated using the following formula: (1 − mortality in control group/mortality in experimental group) × 100. At the end of the infection period, the shrimp hepatopancreas was collected for an RT-qPCR analysis of immune-related genes and histopathological examination. The primers used for the RT-qPCR analysis are listed in Table 3.

### 2.8. Statistical Analysis

The statistical data were analyzed by Graphpad Prism 9 (Graph Pad Software, Inc., San Diego, CA, USA), and significance was analyzed using one-way ANOVA or two-way ANOVA followed by the Duncan’s multiple range test. Each experiment was repeated independently at least three times, and the results are expressed as mean ± SD. Differences were considered to be significant if *p* < 0.05 (* *p* < 0.05, ** *p* < 0.01, and *** *p* < 0.001).

## 3. Results

### 3.1. Construction of the msbB Deletion Mutant and Growth Assessment

**Figure 1:**
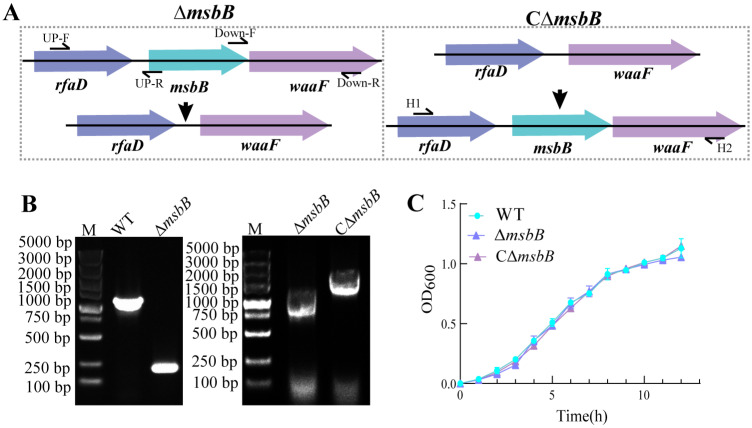
(**A**) The strategy of *msbB* gene was knocked out and complemented by allele exchange in *V. parahaemolyticus*; (**B**) agarose gel electrophoresis of PCR products amplified by Δ*msbB* T1/T2 and CΔ*msbB* T1/T2 separately. Left: WT (1160 bp) and Δ*msbB* mutant (200 bp); right: Δ*msbB* mutant (1352 bp) and its complementary strain CΔ*msbB* (2313 bp); and (**C**) the growth curves of WT, Δ*msbB*, and CΔ*msbB* strains. *n* = 3.

To clarify the role of the *msbB* gene, we inactivated the *msbB* gene by introducing an in-frame deletion. Firstly, the upstream fragment A and downstream fragment B of the *msbB* gene, measuring 632 bp and 757 bp, respectively, were obtained (Figure 1A). Subsequently, the A and B fragments were ligated into the suicide plasmid pSR47S and generated the recombinant plasmid pSR47S-*msbB*. Using suicide-plasmid-mediated homologous recombination, an *msbB* deletion mutation (Δ*msbB*) was constructed. The complementation of Δ*msbB* was achieved using forward screening methods, resulting in the successful construction of CΔ*msbB* (Figure 1B). The deletion of the *msbB* gene in the Δ*msbB* strain and its successful complementation in the CΔ*msbB* strain were confirmed. To assess the necessity of the *msbB* gene for *V. parahaemolyticus*, growth curves were generated for the WT, Δ*msbB*, and CΔ*msbB* strains. As depicted in Figure 1C, all three strains exhibited comparable growth patterns, suggesting that *msbB* is a non-essential gene. Its deletion does not compromise bacterial viability or growth, thus eliminating potential growth discrepancies that could confound subsequent experiments.

### 3.2. Loss of msbB Increased the Sensitivity to Stress and Antibiotics

**Figure 2:**
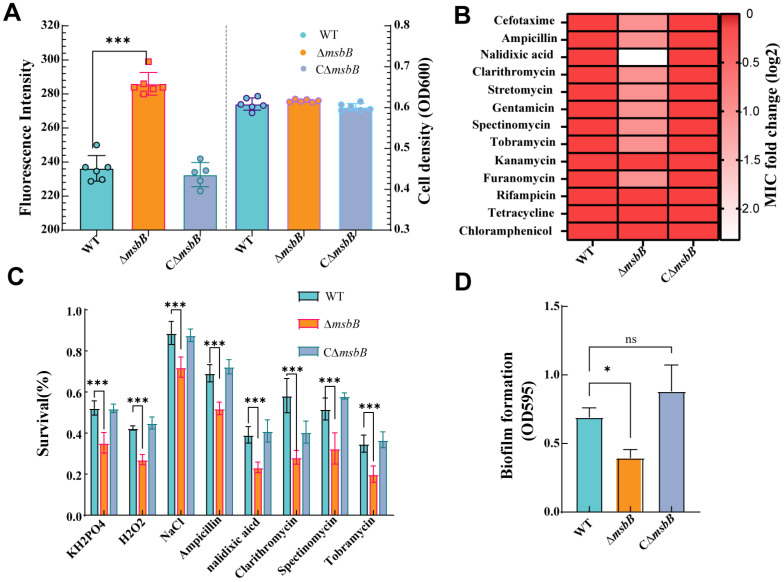
(**A**) Comparison of outer membrane permeability of WT, ∆*msbB* and C∆*msbB*. The fluorescence intensity was determined with excitation at 360 nm and emission at 428 nm. Statistical comparisons were performed using one-way ANOVA analyses followed by a Dunnett’s multiple comparison test; each bar represents as the mean  ±  SD. *n* = 6; (**B**) comparison of resistance to 13 antibiotics among WT, ∆*msbB* and C∆*msbB*. The fold change is calculated by log2 (the MIC of mutants/MIC of WT); (**C**) survival of *V. parahaemolyticus* and Δ*msbB* in the presence of varying different ions and antibiotics. Error bars represent the standard deviation of the mean from at least three (*n* = 3) independent replicates. Statistical comparisons were performed using two-way ANOVA analyses followed by a Dunnett’s multiple comparison test; and (**D**) analysis of biofilm formation ability of WT, ∆*msbB* and C∆*msbB* strains. Statistical comparisons were performed using one-way ANOVA analyses followed by a Dunnett’s multiple comparison test. The data are presented as the mean ± SD (*n* = 3). Columns have been marked with an asterisk (* *p* < 0.05; *** *p* < 0.001). ns: not significant.

The outer membrane permeabilization of the wild-type (WT) and mutant strains was assessed using the N-phenyl-1-napthylamine (NPN) uptake assay. The fluorescence intensity of the Δ*msbB* mutant was significantly higher than that of the WT and complementary strain CΔ*msbB* (Figure 2A), indicating that the deletion of the *msbB* gene increased the outer membrane permeability. Furthermore, the minimal inhibitory concentrations (MICs) of β-lactam antibiotics (cefotaxime 1 µg/mL and ampicillin 8 µg/mL), quinolone antibiotics (nalidixic acid 0.05 µg/mL), macrolide antibiotics (clarithromycin 25 µg/mL), and aminoglycosides (streptomycin 6.25 µg/mL and tobramycin 1.25 µg/mL) for the Δ*msbB* mutant were two-fold lower than those for the WT and CΔ*msbB* strains (Figure 2B). In contrast, the MICs of rifampicin, tetracycline, and chloramphenicol for the Δ*msbB* mutant were similar to those of the WT and CΔ*msbB* strains (Figure 2B). Under stress conditions involving KH_2_PO_4_, NaCl, and H_2_O_2_, the Δ*msbB* strain exhibited the lowest optical density at 600 nm (OD_600_) compared to the WT and CΔ*msbB* strains (Figure 2C). Similarly, when exposed to various antimicrobial compounds, the Δ*msbB* strain showed a significantly inhibited growth compared to WT and CΔ*msbB* (Figure 2C). These results indicate that the Δ*msbB* strain had a lower resistance to these stress factors and antibiotics. Biofilm formation was also evaluated via crystal violet staining, revealing impaired biofilm production in the ∆*msbB* mutant (Figure 2D). These results indicate that the deletion of *msbB* increased the outer membrane permeability, diminished biofilm formation, and led to a reduction in resistance to some ions and antibiotics.

### 3.3. Loss of msbB Reduced the Swarming Motility of V. parahaemolyticus

**Figure 3:**
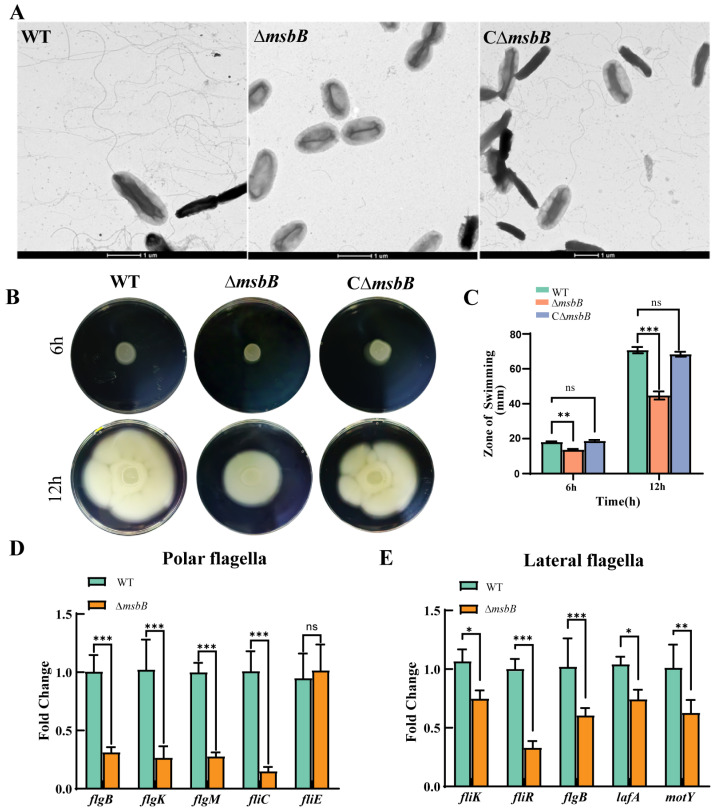
(**A**) Visualization of bacterial flagellar with negative-staining electron microscopy. Scale bar = 1 μm; (**B**) swimming motility assay of WT, ∆*msbB*, and C∆*msbB* on LB plates with 0.3% agar cultured for 6 h and 12 h at 37 °C; (**C**) analysis of motility ability of WT, ∆*msbB*, and C∆*msbB* strains. The diameters of swimming zone reflect bacterial migration on the 0.3% agar. Statistical comparisons were performed using two-way ANOVA analyses followed by a Dunnett’s multiple comparison test; (**D**) qRT-PCR analysis of the transcription levels of polar flagellar genes (*flgB*, *flgK*, *flgM*, *fliC*, and *fliE*) in ∆*msbB* compared to WT. *** *p* < 0.001; and (**E**) qRT-PCR analysis of the transcription levels of lateral flagellar genes (*fliK*, *fliR*, *flgB*, *lafA*, and *motY*) in ∆*msbB* compared to WT. Statistical comparisons were performed using two-way ANOVA analyses followed by a Dunnett’s multiple comparison test; The data are presented as the mean  ±  SD (*n*  =  3). * *p* < 0.05; ** *p* < 0.01; and *** *p* < 0.001. ns: not significant.

First, we visualized the flagella of the WT, Δ*msbB*, and CΔ*msbB* strains using negative-staining electron microscopy. While WT and CΔ*msbB* cells exhibited multiple flagella, the Δ*msbB* strain produced fewer flagella (Figure 3A). To assess motility, we compared the swimming abilities of these strains. The WT and CΔ*msbB* strains spread asymmetrically across the plate, indicative of swimming motility (Figure 3B,C). In contrast, the Δ*msbB* strain showed minimal movement beyond the initial inoculum, suggesting a defect in its swimming motility (Figure 3B,C). We further evaluated the mRNA expression levels of flagellar-related genes via qRT-PCR. Notably, all polar flagellar genes (*flgB*, *flgK*, *flgM*, and *fliC*) and lateral flagellar genes (*fliK*, *fliR*, *flgB*, *lafA*, and *motY*) were significantly downregulated in the Δ*msbB* strain compared to the WT strain. However, the polar flagellar cluster II gene *fliE* showed no significant difference between the WT and Δ*msbB* strains (Figure 3D). These findings confirm that the Δ*msbB* strain had an impaired motility.

### 3.4. The Virulence of ∆msbB Was Attenuated to Tetrahymena

**Figure 4:**
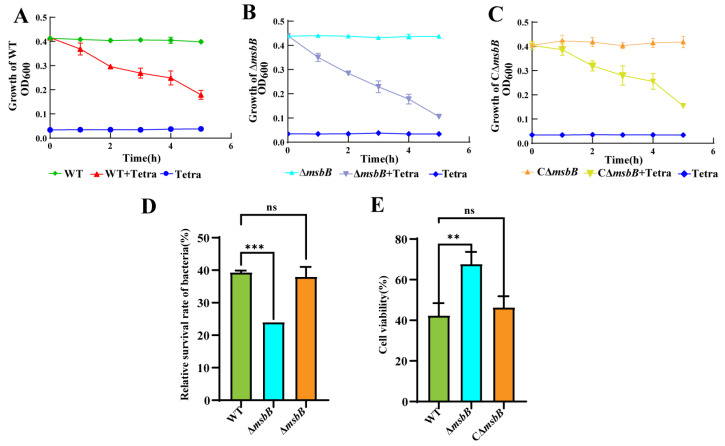
Virulence assessment of WT, ∆*msbB*, and C∆*msbB* using the *Tetrahymena* model. (**A**–**C**) Growth dynamics of ∆*msbB*, C∆*msbB*, and WT strains co-cultured with or without *Tetrahymena*. Tetra represents *Tetrahymena*. (**D**) Relative survival of ∆*msbB*, C∆*msbB*, and WT strains co-cultured with *Tetrahymena*. Relative survival was calculated as the OD_600_ of strains co-cultured with *Tetrahymena* divided by the OD_600_ of bacteria grown alone at the end of the experiment. Data are presented as mean ± SD from three independent measurements. *** *p* < 0.001. ns: not significant. (**E**) Viability of *Tetrahymena* cells treated with lysates from the three strains after 6 h, assessed using CCK8 reagents. Statistical comparisons were performed using one-way ANOVA analyses followed by a Dunnett’s multiple comparison test; Data are presented as the mean ± SD (*n* = 3). The significant difference in the results was analyzed. ** *p* < 0.01. ns: not significant.

To assess the virulence of the ∆*msbB* strain, a co-culture experiment was performed with *Tetrahymena*. In co-culture experiments with *Tetrahymena*, the Δ*msbB* strain was phagocytosed at a significantly faster rate compared to the wild-type (WT) and complementary Δ*msbB* (CΔ*msbB*) strains. After 6 h of co-culture, OD_600_ of the Δ*msbB* strain was the lowest among the three strains, with values of 0.1 for Δ*msbB*, 0.21 for WT, and 0.23 for CΔ*msbB* (Figure 4A–D). This indicates that the Δ*msbB* strain had a reduced ability to resist phagocytosis by *Tetrahymena*. Furthermore, to assess the impact of *msbB* deletion on cellular viability, lysates from the three strains were used to treat *Tetrahymena*, and cell viability was assessed using the Cell Counting Kit-8 (CCK-8) assay. The results showed that the *Tetrahymena* treated with Δ*msbB* lysate had a higher viability compared to those treated with WT and CΔ*msbB* lysates (Figure 4E). This suggests that the deletion of *msbB* not only affected the bacteria’s resistance to phagocytosis, but also reduced its cytotoxicity towards *Tetrahymena*. These findings collectively indicate that the deletion of the *msbB* gene significantly reduced the virulence of the bacteria.

### 3.5. Attenuated Pathogenicity of ΔmsbB Strain Against Shrimp

**Figure 5:**
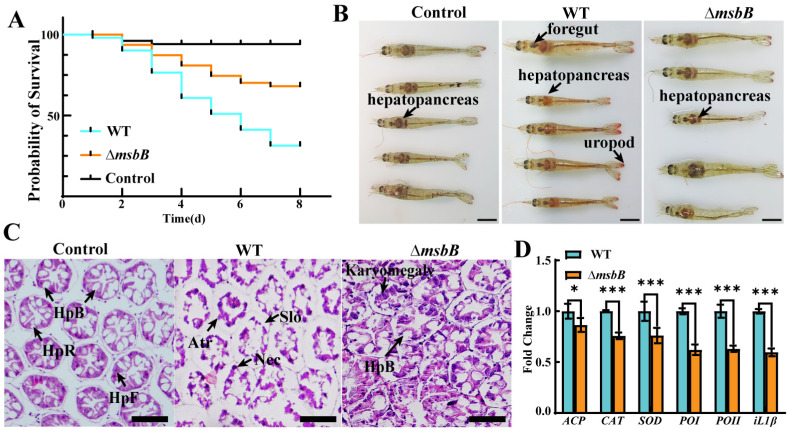
Assessment of ∆*msbB* and WT strains’ pathogenicity against shrimp. (**A**) Relative survival curve of shrimp infected by ∆*msbB* and WT strains with immersion in concentration 4 × 10^7^ CFU/mL; (**B**) clinical sign of diseased shrimps during infection at 10 d. The arrows indicate hepatopancreas, foregut, and uropod. Scale bar: 1 cm; (**C**) hepatopancreas of shrimp challenged with ∆*msbB* and WT strains at 7 d stained with H&E. HpB, B cells; HpF, F cell; HpE, E cell; HpR, R cell; karyomegaly, enlargement of nucleus; Nec, necrosis; Atr, atrophy; Slo, sloughing of epithelial cells. Scale bar: 100 μm; and (**D**) comparative analysis of the expression of immune-related genes and inflammatory cytokine interleukin-1β (*IL-1β*) gene in hepatopancreas of shrimp infected with ∆*msbB* strain vs. WT group. Statistical comparisons were performed using two-way ANOVA analyses followed by a Dunnett’s multiple comparison test; Columns have been marked with an asterisk (* *p* < 0.05; *** *p* < 0.001).

Infection experiments were conducted to assess the lethality and pathogenicity of Δ*msbB* in *L. vannamei* shrimp. The relative percent survival (RPS) of shrimp infected with ∆*msbB* was 78%, while the relative percent survival (RPS) of shrimp infected with the WT was 38% (Figure 5A). As depicted in Figure 5B, the shrimp infected with the WT showed reddening of the uropod, an enlarged stomach, and yellow discoloration of the hepatopancreas, whereas the group infected with the ∆*msbB* strain displayed a slightly yellow discoloration of the hepatopancreas at the late stage of infection. Histopathological sections were prepared to evaluate the impact of Δ*msbB* infections on the shrimp hepatopancreas. The histopathology of the shrimp hepatopancreas revealed that uninfected shrimps exhibited a normal hepatopancreas structure with a high number of B cells, R cells, and F cells (Figure 5C). In contrast, the H&E staining of infected shrimp showed histopathological changes in hepatopancreatic tissues due to bacterial infection. Shrimp infected with the WT strain exhibited sloughing of the epithelial cells (Slo), structural atrophy, and necrosis (Nec) (Figure 5C). In contrast, the ∆*msbB* group exhibited a normal structure and a reduction in hepatopancreas injury, but sloughing of the epithelial cells (Slo) and karyomegaly were evident. The relative expression levels of immune-related genes, such as *ACP*, *SOD*, *CAT*, *PO1*, *PO2*, and inflammatory cytokine interleukin-1β (*IL-1β*), were tested in shrimp hepatopancreas infected with the WT and Δ*msbB*. The expressions of all immune genes and the inflammatory cytokine interleukin-1β (*IL-1β*) gene were significantly lower in the groups treated with Δ*msbB* than in the WT group (Figure 5D). Taken together, these results demonstrate a significantly attenuated pathogenicity of ∆*msbB* in vivo.

## 4. Discussion

This study investigated the role of the *msbB* gene in *V. parahaemolyticus* by constructing a deletion mutant (Δ*msbB*) and a complementary strain (CΔ*msbB*). Our results demonstrated that the deletion of *msbB* increased outer membrane permeability, reduced biofilm formation, and diminished swarming motility. Furthermore, the Δ*msbB* strain exhibited attenuated virulence in both *Tetrahymena* and shrimp infection models, indicating a reduced ability to resist phagocytosis and a decreased cytotoxicity.

The *msbB* gene has been extensively investigated in various Gram-negative bacteria, including *E. coli*, *N. meningitidis*, and *S. enterica* [27,28,29,30,31]. In *E. coli*, the deletion of the *msbB* gene impairs lipid A synthesis, thereby compromising the integrity of the outer membrane [27].

In our study, the deletion of the *msbB* gene resulted in a significant enhancement of NPN fluorescence signals, indicating that NPN could more readily penetrate the cell interior. This suggests an increase in outer membrane permeability. Gram-negative bacteria are characterized by their outer membrane (OM), which functions as an asymmetric barrier. The inner leaflet of the OM is predominantly composed of glycerophospholipids, whereas the outer leaflet is made up of lipopolysaccharide (LPS) [35]. Lipid A, the lipid anchor of LPS, constitutes the outer leaflet of the OM and is essential for maintaining its structural integrity. The *msbB* gene encodes a myristoyltransferase that catalyzes the final step of lipid A biosynthesis [36,37]. In *Klebsiella pneumoniae*, the deletion of the *msbB* gene, which is involved in a similar pathway, results in an increased outer membrane permeability [38]. Consistent with this, our results demonstrated that the deletion of the *msbB* gene significantly enhanced outer membrane permeability. This evidence suggests that the *msbB* gene may influence the structural integrity of the outer membrane, primarily through its role in lipid A biosynthesis.

Antibiotic resistance has become a major global health challenge, with multidrug-resistant bacteria posing significant threats to the effective treatment of infections [39,40]. In recent years, the problem of antibiotic resistance in *V. parahaemolyticus* has become increasingly severe due to the overuse and misuse of antibiotics [1,41]. Research has demonstrated that the resistance mechanisms of *V. parahaemolyticus* are multifaceted, involving horizontal gene transfer, alterations in cell membrane structure, efflux pump systems, and the formation of biofilms, among other factors [1,42,43].

To date, most antibiotics are designed to target intracellular processes and must penetrate the bacterial cell envelope to exert their effects [44]. For Gram-negative bacteria, including *Vibrio parahaemolyticus*, the outer membrane acts as a primary barrier against antibiotic entry. This membrane is composed of lipopolysaccharide (LPS) and glycerophospholipids, and the synchronized synthesis of these components is essential for maintaining the unique permeability barrier of the outer membrane [45]. Impaired outer membrane integrity increases bacterial sensitivity to environmental stress and antibiotics [44,46]. In our study, the Δ*msbB* mutant exhibited an increased outer membrane permeability and sensitivity to stress factors and antibiotics. This finding is consistent with observations in other Gram-negative bacteria, such as *K. pneumoniae*, where deletions in lipid A biosynthesis pathways similarly lead to an increased outer membrane permeability and heightened antibiotic sensitivity [38]. Our results indicate that an increased sensitivity to certain antibiotics in the *msbB* mutant is associated with an altered outer membrane permeability.

Moreover, our study showed that the Δ*msbB* mutant exhibited a reduced capacity for biofilm formation. To date, no reports have elucidated the molecular mechanisms by which the *msbB* gene impacts biofilm formation. Flagella and motility are key factors in the initiation and maturation of biofilms [47]. Our results corroborate this theory, as *msbB* mutants exhibited a reduced motility and biofilm formation. We speculate that the impaired biofilm formation in Δ*msbB* mutants may primarily be associated with defective flagella synthesis. Moreover, biofilm formation and stability are influenced by a multitude of factors, including environmental stress, nutritional conditions, and host immune responses [48]. We hypothesize that changes in biofilm characteristics may also be related to the compromised outer membrane integrity caused by *msbB* deletion, leading to imbalances in material transport capabilities. Further investigation is required to elucidate the detailed molecular mechanisms by which the *msbB* gene affects biofilm formation. Some bacteria form biofilms to reduce antibiotic penetration; biofilms are complex bacterial communities with high levels of drug resistance and environmental adaptability [48,49]. The enhanced antibiotic sensitivity of our Δ*msbB* mutant may be attributed not only to a compromised biofilm integrity, but also to its impaired biofilm formation capability. In summary, these phenomena suggest that the role of the *msbB* gene in maintaining both outer membrane stability and biofilm formation is conserved across different Gram-negative bacterial species.

We found that the deletion of *msbB* significantly inhibited bacterial motility. Transmission electron microscopy (TEM) revealed that flagella synthesis was affected in the Δ*msbB* mutant. Compared to the WT, the mutant exhibited a significant reduction in both the number and morphology of lateral and polar flagella. Since flagella are the primary organelles responsible for bacterial motility, these findings indicate that *msbB* influences bacterial motility by affecting flagella synthesis. Quantitative PCR (qPCR) analysis showed that the expression levels of genes controlling both polar and lateral flagella were significantly downregulated in the Δ*msbB* mutant. This suggests that *msbB* deletion affects the expression of genes related to flagella synthesis, thereby inhibiting flagella formation. Although few studies have reported on the impact of msbB on bacterial motility, it has been demonstrated that flagella assembly in *E*. *coli* depends on the length of lipopolysaccharide (LPS) [50]. Given that *msbB* is involved in LPS synthesis, the influence of *msbB* on motility may also be related to LPS length. However, the specific regulatory molecular mechanisms of this require further investigation.

The attenuation of bacterial endotoxins is a critical factor in reducing the pathogenicity of bacteria towards hosts. In a murine model, *msbB*-deleted *S.typhimurium* exhibited significant attenuation and was capable of eliciting a robust immune response, including the production of IgG and IgA, as well as the secretion of cytokines [30]. Previous studies have shown that LPS produced by *msbB*-deleted *E*. *coli* has a significantly reduced ability to activate an inflammatory response in the host [51]. Similarly, *msbB*-deleted *S.typhimurium* elicits a reduced inflammatory response [30,31].

Our study demonstrated that the deletion of the *msbB* gene in *V. parahaemolyticus* significantly reduced the mortality rate in shrimp, alleviated hepatopancreatic lesion symptoms, and notably decreased the expression of the inflammatory cytokine interleukin-1β (*IL-1β*) gene. The *IL-1β* gene can enhance the phagocytic function of immune cells and stimulates the production of more inflammatory mediators, creating a cascade effect [52,53]. This suggests that the Δ*msbB* strain causes a decreased inflammatory response to shrimp. Vaccine development based on the *msbB* gene has shown promising results. Researchers have utilized *msbB*-deleted bacteria or their LPS as vaccine antigens to induce immune protection in hosts [31]. In animal experiments, vaccines made from *msbB*-deleted bacteria effectively protected animals from infection by wild-type bacteria, demonstrating a good immunogenicity and protective efficacy [31,54]. This indicates that *msbB*-deleted bacteria can also serve as effective vaccine candidates in *V. parahaemolyticus*, providing a novel approach to combat bacterial infections.

The *msbB* gene of *V. parahaemolyticus* is involved in the synthesis of endotoxins, and its deletion leads to a significant reduction in endotoxin activity. Endotoxins are an essential component of the bacterial cell wall and can be recognized by the host immune system to trigger an immune response [24,27,29]. Therefore, the reduced endotoxin activity of the Δ*msbB* strain may diminish the activation of the shrimp immune system.

In our study, although the immune response in shrimp infected with *msbB*-deleted *V. parahaemolyticus* was lower than that in shrimp infected with the wild-type (WT) strain, an immune response was still observed. Among the immune-related genes, the expression level of the acid phosphatase (ACP) gene decreased the least, with a reduction of less than 20%. *Vibrio* species infection typically activates the expression of various immune-related genes in shrimp, such as *ACP*, *SOD*, *CAT*, *PO1*, and *PO2*. ACP functions by hydrolyzing components of the pathogen cell wall, while *SOD* and *CAT* work by scavenging reactive oxygen species to reduce the damage caused by pathogens to the cells. *PO1* and *PO2*, on the other hand, synthesize melanin to encapsulate pathogens, thereby preventing their spread [55,56]. These immune-related genes work together through multiple mechanisms to enhance the immune defense capabilities of shrimp. Overall, Δ*msbB* still elicited a high level of immune response to *V. parahaemolyticus*, while significantly reducing its pathogenicity in shrimp. This suggests that *msbB* gene deletion can modulate the host’s immune response, reducing the severity of infection without completely abolishing the immune reaction.

## 5. Conclusions

This study investigated the role of the *msbB* gene in *V. parahaemolyticus* using a deletion mutant (Δ*msbB*). The results showed that *msbB* deletion increased outer membrane permeability, reduced biofilm formation, and diminished swarming motility. The Δ*msbB* strain exhibited attenuated virulence in shrimp, with a reduced mortality and fewer hepatopancreatic lesion symptoms. These findings align with studies in other Gram-negative bacteria, highlighting *msbB*’s role in maintaining outer membrane stability and biofilm formation. The study also suggests that *msbB* deletion can modulate the host’s immune response, reducing pathogenicity while still eliciting an immune reaction. This indicates the potential of *msbB*-deleted bacteria as vaccine candidates. However, the study was limited by the lack of long-term infection models to assess the durability of the attenuated phenotype and the immune response. Future studies should address these limitations to fully realize the vaccine potential of *msbB*-deleted strains.

## Figures and Tables

**Table 1:** Strains and plasmids.

Strains	Genotype and Characteristics	Source
*V. Parahaemolyticus 17802*	Cms, Kms, Ampr, Wild-type strain	ATCC
∆*msbB*	*V. parahaemolyticus* strain in-frame deletion in *msbB*	This study
C∆*msbB*	The complement of ∆*msbB*	This study
*Escherichia coli*		
CC118	λpir lysogen of CC118 (Δ(ara-leu) araD ΔlacX74galEgalKphoA20 thi-1rpsE rpoB argE (Am) recA1	Our lab
CC118/pHelper	CC118 λpir harboring plasmid pHelper	Our lab
Plasmids		
pSR47S	Bacterial allelic exchange vector with sacB, KanR	Our lab
pSR47S-∆*msbB*	A 1394 bp fragment encompassing the upstream and downstream regions of the *msbB* gene in pSR47S, KanR	This study
pSR47S-C∆*msbB*	A 2313 bp fragment harboring the *msbB* sequences in pSR47S, KanR	This study

**Table 2:** Sequences of PCR oligonucleotide primers.

Primer Name	Primer Sequence (5′ to 3′)	Purpose
UP-F	GCCGCTCTAGAACTAGTGGATCTAACTACGT TCGTCGTCTATGGC	Creation of ∆*msbB* deletion fusion fragment
UP-R	CAACTTGCCAGTCAGTGTTGTAGCGTGTCTAT TACCTATCCGTC	
DOWN-F	GACGGATAGGTAACTAGACACGCTACAACACTGACTGGCAAGTTG	
DOWN-R	GGATCGATCCTCTAGATCGATGTGTTCA TGGAACTGTGTTGG	
*msbB*-H1	CTCTAGAACTAGTGGATCCGTTGAAGAACGCGAATACGAAG	Creation of C∆*msbB* fragment
*msbB*-H2	TCGATCCTCTAGAGTCGAGTGCAGCAACTT CGGCATAGTG	
∆*msbB-*T1	GTAGCATCACTTGTTGCCACC	Confirmation of deletion strain
∆*msbB-*T2	GGACATCACCATATCACCAACC	
C∆*msbB-*T1	CGTTGAAGAACGCGAATACGAAG	Confirmation of complementary strain
C∆*msbB-*T2	GTGCAGCAACTTCGGCATAGTG	

Note: Complementary sites are underlined.

**Table 3:** Primers used for qRT-PCR.

Primer Name	Primer Sequence (5′ to 3′)
Liva *proPOI*-F	ACGTCACTTCCGGCAAGCGA
Liva *proPOI*-R	CCTCCTTGTGAGCGTTGTCAGG
Liva *proPO II*-F	ACCACTGGCACTGGCACCTCGTCTA
Liva *proPO II*-R	TCGCCAGTTCTCGAGCTTCTGCAC
Liva *cytMnSOD*-F	TGACGAGAGCTTTGGATCATTCC
Liva *cytMnSOD*-R	TGATTTGCAAGGGATCCTGGTT
*CAT*-F	ATCCATTCGACCTTACCA
*CAT*-R	ACGCAATCTGCTCCACCT
*ACP*-F	GTAGCATCACTTGTTGCCACC
*ACP*-R	GGACATCACCATATCACCAACC
*IL-1β*-F	CATCCCATTTGTGGTTCTG
*IL-1β*-R	TCGTGCTTCACTATGCCTC
*β-actin*-F	AGTAGCCGCCCTGGTTGT
*β-actin*-R	AGGATACCTCGCTTGCTCT

## Data Availability

The data that support the findings of this study are available from the FigShare at 10.6084/m9.figshare.28241171 or the corresponding author upon reasonable request.
